# Association of Single Nucleotide Polymorphisms of Calcium-Sensing Receptors With the Occurrence and Recurrence of Nephrolithiasis in the Indian Population

**DOI:** 10.7759/cureus.85313

**Published:** 2025-06-03

**Authors:** Madhura NS, Kowsalya R, Shivakumar V

**Affiliations:** 1 Biochemistry, Institute of Nephro-Urology, Bengaluru, IND; 2 Urology, Institute of Nephro-Urology, Bengaluru, IND

**Keywords:** calcium-sensing receptor, genetic association, genotyping, kidney stone disease, single nucleotide polymorphism

## Abstract

Background

Nephrolithiasis, commonly known as kidney stone disease (KSD), is a multi-factorial disorder influenced by genetic and metabolic factors. The calcium-sensing receptor (CASR) plays a crucial role in calcium homeostasis, and single-nucleotide polymorphisms (SNPs) in the CASR gene may contribute to the occurrence and recurrence of nephrolithiasis. This study investigates the association of CASR SNPs (rs1801725, rs1042636, and rs1501899) with KSD in the Indian population.

Methods

A single Institute case-control study was conducted between May 2022 and May 2024, including 110 KSD patients and 100 healthy controls. Genotyping of CASR polymorphisms was performed using TaqMan®SNP Genotyping Assay. The association between SNPs and KSD risk was evaluated using odds ratios.

Results

The T allele of SNP rs1801725 and the G allele of SNP rs1042636 were significantly associated with an increased risk of KSD (P < 0.0001) and were linked to hypercalcemia and hypercalciuria. The A allele of SNP rs1501899 showed a weaker, non-significant association with KSD (P = 0.0767). The G allele of SNP rs1042636 showed a potential trend toward increased risk of recurrent stone formation (OR = 1.87, p = 0.113).

Conclusion

Our findings suggest that CASR polymorphisms rs1801725 and rs1042636 contribute to KSD susceptibility in the Indian population, influencing calcium metabolism. Genetic screening for these SNPs may aid in early detection and personalized management of KSD patients, particularly those at risk for recurrent stones.

## Introduction

Nephrolithiasis, commonly referred to as kidney stone disease (KSD), is a common health problem worldwide with a lifetime risk of 12% in men and 6% in women. The global prevalence of kidney stones is estimated to be between 10% and 15%, with some regions experiencing higher rates [1-3]. In India, the incidence of the disease has been on the rise in the last two decades, mirroring global trends [4-5].

KSD is characterized by the formation of hard mineral deposits inside the kidneys, which may cause excruciating pain, hematuria, and urinary obstruction when they move through the urinary tract. In chronic conditions, it can lead to long-term complications, persistent discomfort, and psychological distress [6]. The recurrence rate of the disease is notably high, with up to 50% of patients experiencing another episode within 5 years and 10% facing multiple episodes within 10 years [7]. There is a need for increased awareness, preventive measures, and early detection strategies to manage this growing issue. Knowledge gaps in the genetics of kidney stone disease (KSD), particularly regarding genetic predisposition and its role in emerging therapies, underscore the need for ongoing research.

Single-nucleotide polymorphisms (SNPs), which involve single-base substitutions in the genes, are the most common type of genetic mutation in the human genome [8]. These mutations can impact gene function, protein expression, and metabolic pathways, thereby influencing disease susceptibility. One of the key genes associated with KSD is the calcium-sensing receptor (CaSR) gene, which plays a key role in maintaining calcium homeostasis. CaSR gene (chr. 3q13.3-21) encodes for the CaSR protein of 1078 amino acids present in the plasma membrane as a dimer. CaSR is a member of the G-protein coupled receptors [9,10].

CaSR is expressed in the parathyroid glands and renal tubules, especially the distal tubules [11], and hence, it regulates calcium metabolism by modulating parathyroid hormone secretion and renal calcium reabsorption. Polymorphisms in this gene can disrupt calcium homeostasis, increasing the risk of hypercalciuria and subsequent kidney stone formation. In this context, specific SNPs of CaSR, such as Arg990Gly (rs1042636) and Ala986Ser (rs1801725), have been identified as significant contributors to KSD [12-15].

The genetic variations and hence their influence on disease expression are known to vary across populations [16], highlighting the importance of studying these SNPs in diverse demographic groups with KSD to draw robust conclusions about the genetic predisposition of the disease. This research is particularly significant, as it is being conducted at the only tertiary care center for nephrology and urology in Bengaluru, where a high burden of kidney stone disease is routinely encountered.

The primary objective of the study is to assess the association of SNPs of the CASR gene, particularly rs1801725, rs1042636, and rs1501899, with the risk of KSD in the Indian population. The secondary objectives are to evaluate the potential role of CASR SNPs in the recurrence of KSD and to explore the association of these genetic variants with metabolic abnormalities such as hypercalcemia and hypercalciuria.

## Materials and methods

Study design

This is a single-center case-control study conducted in the Institute of Nephro-Urology, Bangalore, a tertiary care center for nephrology and urology care.

Recruitment of study participants

Cases

Patients with nephrolithiasis (KSD), aged between 18 and 60 years, undergoing surgical or endoscopic stone removal in the urology department of our hospital between May 2022 and May 2024 were identified. Following surgery, the stones removed were analyzed in the biochemistry laboratory by the wet chemical analysis method. Those patients with calcium-containing stones were selected and were provided with detailed information about the study, including its purpose, procedures, and potential risks. Patients who were willing to join the study were then enrolled in the study after obtaining informed consent. Patients on drugs affecting calcium and phosphorus levels (steroids, diuretics, vitamin D, bisphosphonates) were excluded from the study. Other exclusion criteria were patients with endocrine disorders like primary hyperparathyroidism or hyperparathyroidism (affecting calcium levels in the blood) and chronic kidney disease (affecting calcium, introducing heterogeneity). The diagnosis of KSD was made based on renal colic with or without hematuria, with radiographic evidence of a stone. A kidney stone was defined as a calcium stone if it was made of calcium oxalate or calcium phosphate on wet chemical analysis of the stone.

Controls

About 100 control participants were recruited from routine outpatient departments, ensuring that they had no clinical or radiological evidence of KSD or had no history of kidney stones. The age and gender of the controls were matched to the cases. 

Sample Collection and Storage

Blood samples were collected randomly using the standard venipuncture technique into both plain and ethylenediaminetetraacetic acid (EDTA) tubes under aseptic conditions. Samples from EDTA tubes were stored at -20 °C until DNA extraction and later used for genotyping. Samples from the plain tubes were used for other biochemical analyses. These samples were ensured to have complete clot formation before centrifugation. The samples were centrifuged for 15 minutes, and the serum was separated and analyzed on the same day. Kidney stones of patients who underwent surgery were collected from the urology department and stored at room temperature until the analysis.

For 24-hour urine collection, all participants were given a container and were instructed to discard the first morning urine, note the time, and collect all urine for the next 24 hours. The final sample was collected at the same time the next day. The collected urine was then submitted to the lab as instructed on the same day.

Wet chemical analysis of kidney stones: The stones were dried and finely powdered using a mortar and pestle. The powdered sample was then dissolved in appropriate acids, such as hydrochloric acid or nitric acid, for further chemical analysis.

DNA extraction: QIAamp DNA Blood Mini Kits from Qiagen (Hilden, Germany) were used for DNA extraction. This procedure was done in the reverse transcription-polymerase chain reaction (RT-PCR) laboratory of our institute. The method involves the solid-phase extraction principle, where a silica membrane-based spin column protocol is used for extracting DNA. Extraction follows four basic steps: 1) Lysis of the blood cells, 2) Selective binding of DNA on a silicon column, 3) Removal of contaminants by washing, and 4) Elution involving the collection of purified DNA. DNA thus extracted is stored at -20 °C until genotyping.

Genotyping: SNP genotyping was performed by the hydrolysis probe method. The TaqMan®SNP Genotyping Assay (Applied Biosystems Inc., Waltham, USA) was used to analyze rs1801725, rs1042636, and rs1501899 of CASR on the QIAquant 96 5plex real-time qPCR System (Qiagen). Briefly, the assay employs the 5′ nuclease activity of Taq polymerase to generate a fluorescent signal during PCR. For each of the CASR SNPs, two probes were utilized: the wild-type and variant allele probes. A 5′ reporter dye (FAM or VIC) and a 3′ quencher dye (MGB/Black Hole Quencher (BHQ)) were linked to each probe. The nuclease activity only happens with the perfectly hybridized probes. The ratio of the fluorescent signal for the two reporter dyes is measured and used as an indication of the sample genotype.

Stringent quality control measures were implemented throughout the process to ensure the accuracy and reliability of the genotyping data. Negative controls were included in each qPCR run to monitor for contamination. A subset of randomly selected samples (10%) was genotyped in duplicate to assess reproducibility. Genotyping call rates exceeded 95% for all three CASR SNPs (rs1801725, rs1042636, and rs1501899), and samples with ambiguous or failed calls were excluded from the analysis. Additionally, Hardy-Weinberg equilibrium (HWE) was assessed in the control group for each SNP to confirm the quality of the genetic data and population stability.

Biochemical analysis: Serum analysis was performed using the Abbott Alinity chemistry and immunoassay auto-analyzer (Abbott Laboratories, Lake County, Illinois, USA) in the biochemistry laboratory. Ready-to-use kits from Abbott Alinity c and i systems were utilized for the analyses. Blood urea was assayed by the urease method and creatinine by Jaffe's method, total serum calcium by the Arsenazo III dye-binding method, serum 25-hydroxyvitamin D (25-OH D) levels by using chemiluminescent Immunoassay (CLIA), serum uric acid using the urease enzymatic method, serum phosphorus (inorganic phosphate) using the phosphomolybdate UV method.

Statistical analysis

All statistical analyses were performed using IBM SPSS Statistics for Windows, Version 26.0 (IBM Corp., Armonk, NY, USA). Continuous variables were expressed as mean ± standard deviation (SD), and categorical variables were presented as frequencies and percentages. The independent t-test was used to compare means between KSD patients and controls. The chi-square test and the Z-test for proportions were used to assess differences in genotype and allele frequencies between groups. To evaluate the genetic association, odds ratios (ORs) with 95% confidence intervals (CIs) were calculated for different genetic models (dominant, recessive, and allelic). A p-value < 0.05 was considered statistically significant. Genotype models were constructed to explore the dominant (heterozygous + homozygous variant vs. wild type) and recessive (homozygous variant vs. heterozygous + wild type) effects of each SNP. The association of SNPs with recurrence and metabolic parameters was also explored using cross-tabulation and ORs.

Sample size calculation and justification

Based on previous studies, the prevalence of nephrolithiasis in the population (p₀) is estimated at 15% (p₀ = 0.15), with an assumed odds ratio of 3 for the risk factor in cases, giving an estimated proportion of cases with the risk factor as 45% (p₁ = 0.45). Accordingly, q₀ = 1 - p₀ = 0.85 and q₁ = 1 - p₁ = 0.55. Using a conventional significance level (α) of 0.05 (two-sided) and power (1 - β) of 80% (β = 0.20), and assuming equal sample sizes in both groups (n₁ = n₂), the required sample size is calculated using the formula:

$n = \frac{(p_0 q_0 + p_1 q_1)(Z_{1 - \alpha/2} + Z_{1 - \beta})^2}{(p_1 - p_0)^2}$

Substituting the values:

$n = \frac{(0.15 \times 0.85 + 0.45 \times 0.55)(1.96 + 0.84)^2}{(0.45 - 0.15)^2} = 91.6$

Although the minimum required sample size per group was calculated to be 92 participants, we have considered 110 cases and 100 controls to improve statistical power and precision.

## Results

The biochemical and demographic characteristics of all subjects (cases=110, controls=100) enrolled in the study are demonstrated in Table 1. The mean age of KSD patients (42±12 years) was comparable to that of the control group (43±9 years) (P=0.49, not significant). The proportion of males (n=69; 62.8%) and females (n=31; 37.2%) in the KSD group was not significantly different from the control group (P=0.14). Serum total calcium and 24-hour urine calcium were significantly higher in KSD patients (P=0.0001 ) while serum uric acid, phosphorus, and vitamin D levels showed no significant difference between KSD patients and controls (P=0.38, P=0.41, P=0.260, respectively).

**Table 1:** The characteristics of KSD patients and controls KSD: Kidney Stone Disease; p < 0.05 → Statistically significant; continuous variables are expressed as mean±SD; the independent t-test was used for continuous variables and the chi-square test for frequencies

Charecteristics (Reference range)	KSD	Control	P Value
Age (years)	42±12	43±9	0.49
Male	69 (62.8%)	59 (59%)	0.14
Female	31 (37.2%)	41 (41%)	
Serum uric acid (2.6-6mg/dl)	4.9±0.4	4.7±0.7	0.38
Serum calcium (8.4-10.2mg/dl)	10.3±1.3	8.7±0.5	0.0001
Serum phosphorus (2.5-4.7mg/dl)	3.9±1.3	3.8±0.8	0.41
Serum vitamin D (30-50ng/ml)	20±15	18±10	0.26
24hr Urine Calcium (100-300mg/day)	259.2±205	169.2±40	0.0001

Table 2 provides details of three CaSR gene SNPs analyzed in this study. SNP rs1801725 (located at Exon 7) involves a G to T substitution at position 2955 of the CASR gene, resulting in an amino acid change from alanine to serine at position 986 (A986S) in the CaSR protein. SNP rs1042636 (located at Exon 7) involves an adenine to guanine substitution at position 1173 of the CASR gene, leading to a change from glutamic acid to glycine at position 986 (E986G) in the CaSR protein. SNP rs1501899 (located in the intron) involves a guanine to adenine substitution in a non-coding intronic region of the CASR gene and does not result in any amino acid change.

**Table 2:** CaSR SNPs under study SNP: single-nucleotide polymorphism

SNP ID	Nucleotide Variation	Amino Acid Variation
rs1801725	G > T	A986S
rs1042636	A>G	E986G
rs1501899	G>A	No amino acid change

Genotype distribution of rs1801725 (G>T)

Table 3 presents the genotype distribution of rs1801725 (G>T) in KSD patients and controls. The GT genotype (n=52; 47%) in KSD and n=10 (10%) in controls, and the TT genotype (n=23; 21%) in cases and n=4 (4%) in controls showed significantly increased risk (P < 0.0001, P=0.0026, respectively) for the disease while the GG genotype being more common in controls n=86 (86%) showed reduced risk (P < 0.0001) with n=35 (32%) in KSD.

**Table 3:** Genotype distribution for SNP rs1801725 SNP: single-nucleotide polymorphism; KSD: kidney stone disease

Genotype	KSD (%), (n = 110)	Controls (%), (n = 100)	Z Score	P Value
GG	35 (32%)	86 (86%)	-7.93	<0.0001
GT	52 (47%)	10 (10%)	5.91	<0.001
TT	23 (21%)	4 (4%)	-3.656	0.00026

We further evaluated dominant and recessive genetic models for SNP rs1801725 to determine their influence on disease risk (Table 4). The dominant model (GT + TT vs. GG) shows a stronger association with disease risk (OR = 13.1633, P < 0.0001) than the recessive model (TT vs. GT + GG, OR = 6.3448, P < 0.0001). This suggests that carrying at least one T allele (GT or TT) significantly increases disease susceptibility, making T a high-risk variant.

**Table 4:** Interpretation of genetic models for rs1801725 Odds ratio for comparison between cases and controls; Odds ratio >1 indicates a positive association; KSD: kidney stone disease

Genetic Model	Genotypes	KSD (%), n=110	Control (%), n=100	Odds Ratio	P Value
Dominant	GT+TT	75 (68.1%)	14 (14%)	13.1633 (6.5835 to 26.3189)	P < 0.000
	GG	35 (31.8%)	86 (86%)		
Recessive	TT	23 (20.9%)	4 (4%)	6.3448 (2.1105 to 19.0748)	P < 0.000
	GT+GG	87 (79%)	96 (96%)		

Genotype distribution of SNP rs1042636

For SNP rs 1042636, the AA genotype (n=40 (36%) in KSD, n=76 (76%) in controls) showed reduced risk (P < 0.001) for the disease (Table 5). The AG genotype (n=45 (41%) in KSD, n=24 (24%) in controls) showed a moderate disease risk (Z = 2.6055, P = 0.00906). The GG genotype (n=25 (22%) in KSD, n=1 (1% ) in controls) is strongly associated with disease susceptibility (Z = 5.0792, P < 0.0001).

**Table 5:** Genotype distribution for SNP rs1042636 SNP: single-nucleotide polymorphism; KSD: kidney stone disease

Genotype	KSD (%) (n = 110)	Controls (%) (n = 100)	Z Score	P Value
AA	40 (36%)	76 (76%)	-5.76	<0.0001
AG	45 (41%)	23 (23%)	2.605	0.00906
GG	25 (22%)	1 (1%)	5.079	<0.0001

The dominant model (AG + GG vs. AA) shows a strong association with disease risk (OR = 5.5, P < 0.0001), indicating that carrying at least one G allele increases susceptibility. The recessive model (AA vs. AG + GG) is more significant (OR = 29.116, P = 0.0011), suggesting that the GG genotype has a significant role in stone formation (Table 6).

**Table 6:** Interpretation of genetic models for SNP rs1042636 SNP: single-nucleotide polymorphism; KSD: kidney stone disease

Genetic Model	Genotypes	KSD(%) n=110	Controls(%) n=100	Odds Ratio	P Value
Dominant	AG+GG	70 (63.6%)	24 (24%)	5.5417 (3.0374 to 10.110)	P < 0.0001
	AA	40 (36.3%)	76 (76%)		
Recessive	GG	25 (22.7%)	1 (1%)	29.116 (3.8637 to 219.4365)	P 0.0011
	AG+AA	85 (77.2%)	99 (99%)		

Genotype distribution of SNP rs1501899

For SNP rs1501899, the AA genotype (n=29 (26%) in KSD, n=6 (6%) in controls) is significantly associated with an increased risk of KSD, while the GG genotype is protective, and the AG genotype does not show a significant association with the disease (Table 7).

**Table 7:** Genotype distribution for SNP rs1501899 SNP: single-nucleotide polymorphism; KSD: kidney stone disease

Genotype	KSD (%), (n = 110)	Controls (%), (n = 100)	Z Score	P Value
AA	29 (26%)	6 (6%)	3.9547	0.0008
AG	18 (16%)	20 (20%)	-0.6836	0.496
GG	63 (57%)	74 (74%)	-2.5422	0.01108

The dominant model (AG + AA vs GG) shows a strong association with disease risk (OR = 2.12, P < 0.011), indicating that carrying at least one A allele increases susceptibility. The recessive model (GG vs. AG + AA) is more significant (OR = 5.6, P = 0.0003), suggesting that the AA genotype has a significant role in stone formation (Table 8).

**Table 8:** Interpretation of genetic models for SNP rs1501899 SNP: single-nucleotide polymorphism

Genetic Model	Genotypes	Cases	Control	Odds Ratio	P Value
Dominant	AG+AA	47 (42.7%)	26 (26%)	2.123 (1.118 to 3.8119)	P 0.011
	GG	63 (57.2%)	74 (74%)		
Recessive	AA	29 (26.3%)	6 (6%)	5.609 (2.217 to 14.186)	P 0.0003
	AG+GG	81 (73.6%)	94 (94%)		

Allelic distribution

Table 9 demonstrates a detailed view of the allelic distribution of the three SNPs under study. The disease-forming alleles, the T allele of rs1801725 and the G allele of rs1042636, show a strong and significant association with KSD risk (OR = 11.0000, P < 0.0001) and (OR = 11.6972, P < 0.0001), respectively. However, the A allele of rs1501899 does not show a significant association with disease risk (OR = 1.8309, P = 0.0767).

**Table 9:** Allelic distribution of the SNPs under study SNP: single-nucleotide polymorphism; KSD: kidney stone disease

SNP ID	Alleles	KSD (%), n=110	Controls (%), N=100	Odds ratio	P value
rs1801725	T	66 (60%)	12 (12%)	11.0000 (5.3884 to 22.4558)	< 0.0001
	G	44 (40%)	88 (88%)		
rs1042636	G	59 (53.6%)	9 (9%)	11.6972 (5.3583 to 25.535)	< 0.0001
	A	51 (46.3%)	91 (91%)		
rs1501899	A	30 (27.2%)	17 (17%)	1.8309 (0.9373 to 3.576)	0.0767
	G	80 (72.7%)	83 (83%)		

The distribution of alleles for three SNPs - rs1801725, rs1042636, and rs1501899 - was compared (Table 10) between new stone formers (NSF) and recurrent stone formers (RSF). SNP rs1042636 (G allele) showed a possible association with recurrent stone formation (OR = 1.87, p = 0.113), though not statistically significant. SNP rs1801725 (T allele) and SNP rs1501899 (A allele) also showed higher frequency in recurrent cases but with no significant association (p = 0.26 and 0.469, respectively).

**Table 10:** Allelic distribution of SNPs in new stone formers and recurrent stone formers NSF: new stone formers, RSF: recurrent stone formers, SNP: single-nucleotide polymorphism

SNP ID	Alleles	NSF n=43	RSF n=67	Odd s ratio	P value
rs1801725	T	23 (53.4%)	43 (64.1%)	1.55 (0.7141 to 3.3989)	0.26
	G	20 ((46.5%)	24 (35.8%)		
rs1042636	G	19 (44.1%)	40 (59.7%)	1.87 (0.862 to 4.0618)	0.113
	A	24 (55.8%)	27 (40.2%)		
rs1501899	A	15 (34.8%)	28 (41.7%)	1.34 (0.606 to 2.962)	0.469
	G	28 (65.1%)	39 (58.2%)		

In the present study, we also found that the disease-forming alleles of the SNPs under study (T in rs1801725, G in rs1042636, and A in rs1501899) showed possible metabolic abnormalities in KSD patients. Among 66 KSD patients carrying the T allele of rs1801725, hypercalcemia was observed in n=22 (33%) and hypercalciuria in n=15 (23%), with no association found with hyperuricemia or hyperphosphatemia. In 59 patients with the G allele of rs1042636, hypercalcemia occurred in n=21 (35%) and hypercalciuria in n=18 (30%). The A allele of rs1501899 showed minimal metabolic impact.

## Discussion

This study aimed to investigate the association of CASR gene polymorphisms (rs1801725, rs1042636, and rs1501899) with KSD and related metabolic abnormalities. Our findings indicate a significant correlation between specific alleles of these polymorphisms and an increased risk of KSD, as well as their impact on metabolic abnormalities such as hypercalcemia, hypercalciuria, and hyperparathyroidism.

Epidemiology of KSD

KSD is a prevalent urological disorder affecting approximately 10-15% of the global population. KSD is a significant health concern with a 12% prevalence rate and a 50% recurrence rate among the Indian population [4-5]. Studies have consistently demonstrated that men have a higher risk of developing kidney stones than women, with a reported male-to-female ratio of approximately 2:1 [1-3]. Our study findings showed a similar pattern, with men (62%) approximately twice as likely as women (37%) to develop kidney stones.

Association of CASR SNPs with KSD risk

Several studies have investigated the role of CASR gene polymorphisms in kidney stone disease (KSD) [12-14]. A study by Vezzoli et al. (2011) reported that the Arg990Gly polymorphism (rs1042636) in the CASR gene is associated with an increased risk of nephrolithiasis, with the GG genotype of rs1042636 having a higher predisposition to developing kidney stones [14]. Similarly, in a study conducted in Eastern India, Guha et al. (2015) found that both rs1801725 and rs1042636 polymorphisms were significantly associated with an increased risk of KSD [15].

In the present study, rs1801725 and rs1042636 were significantly associated with an increased risk of KSD. The dominant and recessive genetic models further confirmed that individuals carrying at least one risk allele had a higher susceptibility to the disease. The dominant model for rs1801725 showed an odds ratio of 13, indicating the individuals with at least one T allele (GT or TT) are 13 times more likely to have the disease than those with GG. Similarly, the dominant model for rs1042636 showed an odds ratio of 6, indicating the individuals with at least one G allele (AG or GG) are six times more likely to have the disease than those with AA. The T allele of rs1801725 (OR: 11.0000, P < 0.0001) and the G allele of rs1042636 (OR: 11.6972, P < 0.0001) were significantly overrepresented in KSD cases, reinforcing their role as a risk allele for KSD.

In contrast, the A allele of rs1501899 showed a weaker, non-significant association with KSD (OR: 1.8309, P = 0.0767), suggesting a minimal impact on disease risk compared to the other two SNPs. This could be attributed to its location within an intronic region of the CASR gene. Unlike exonic polymorphisms (rs1801725 and rs1042636 in the present study), intronic variants are known to have no role in the alteration of protein structure or function, and hence have subtler effects. However, they may still influence gene expression through regulatory mechanisms [17].

Metabolic impact of risk alleles

A genome-wide meta-analysis found that each rs1801725 T allele increases serum calcium by approximately 0.01874 mmol/L, accounting for 1.26% of the variance in serum calcium levels [18-19]. In the present study, a significant proportion of KSD patients carrying the T allele of rs1801725 exhibited hypercalcemia (33%) and hypercalciuria (23%), suggesting that this variant may contribute to altered calcium homeostasis. Similarly, the G allele of rs1042636 was associated with hypercalcemia (35%) and hypercalciuria (30%), reinforcing its role in calcium metabolism and kidney stone formation. Conversely, the A allele of rs1501899, located in a non-coding intronic region, showed minimal impact on metabolic abnormalities, indicating a weaker or non-significant role in disease pathogenesis.

Clinical implications

Identifying individuals carrying these risk alleles may help in early screening and better management strategies for KSD. Patients with rs1801725 (T allele) or rs1042636 (G allele) may benefit from targeted interventions, including dietary modifications and pharmacological approaches, to regulate calcium metabolism and reduce stone formation risk.

Limitations and future directions

While this study provides valuable insights, it has some limitations. The sample size is relatively small, and larger multicenter studies are needed to validate these findings. Furthermore, functional studies are required to elucidate the precise mechanisms by which CASR polymorphisms contribute to calcium dysregulation and stone formation.

## Conclusions

In conclusion, our study highlights a strong association between CASR gene polymorphisms and KSD risk, particularly for rs1801725 (T allele) and rs1042636 (G allele). Although no statistically significant association was found between these SNPs and recurrence, the observed trends in recurrent cases point toward a possible genetic influence requiring further investigation. These variants are linked to significant metabolic abnormalities, including hypercalcemia and hypercalciuria, reinforcing their role in disease pathogenesis. Genetic screening for these polymorphisms may serve as a valuable tool for early detection and personalized management of KSD patients. Further research is warranted to explore the underlying molecular mechanisms and potential therapeutic interventions targeting CASR-related pathways. 
